# Predictors of hepatitis C treatment outcomes in a harm reduction-focused primary care program in New York City

**DOI:** 10.1186/s12954-021-00486-4

**Published:** 2021-03-31

**Authors:** Jacob Ziff, Trang Vu, Danielle Dvir, Farah Riazi, Wilma Toribio, Scott Oster, Keith Sigel, Jeffrey Weiss

**Affiliations:** Respectful and Equitable Access to Comprehensive Healthcare (REACH) Program, Division of General Internal Medicine, Icahn School of Medicine At Mount Sinai, 17 East 102nd Street, 7th floor, New York City, NY 10029 United States

**Keywords:** Hepatitis C, Unstable housing, People who inject drugs, Medicaid, Harm reduction, Direct-acting antiviral

## Abstract

**Background:**

The social determinants of health that influence steps in the entire Hepatitis C Virus (HCV) treatment cascade must be identified to achieve HCV elimination goals. This project aimed to evaluate the association of these factors with HCV treatment completion and return for sustained virologic response (SVR) testing.

**Methods:**

We used retrospective cohort data from our primary care-based HCV treatment program that provides comprehensive harm reduction care to those who use or formerly used drugs. Among persons who began direct-acting antiviral HCV treatment between December 2014 and March 2018, we identified two outcomes: HCV treatment completion and return for SVR assessment 12 weeks after treatment end. Several predictors were ascertained including sociodemographic information, substance use, psychiatric symptoms and history, housing instability, and HCV treatment regimen. We then evaluated associations between predictors and outcomes using univariate and multivariable statistical methods.

**Results:**

From a cohort of 329 patients treated in an urban primary care center, multivariable analysis identified housing instability as a single significant predictor for HCV treatment completion (odds ratio [OR]: 0.3; 95% confidence interval [CI]: 0.1–0.9). Among patients completing treatment, 226 (75%) returned for SVR assessment; the sole predictor of this outcome was Medicaid as primary insurance (compared to other insurances; OR 0.3; 0.1–0.7).

**Conclusions:**

Innovative strategies to help unstably housed persons complete HCV treatment are urgently needed in order to reach HCV elimination targets. Educational and motivational strategies should be developed to promote individuals with Medicaid in particular to return for SVR viral load testing, a critical post-treatment component of the HCV treatment cascade.

*Trial registration* Not applicable.

## Introduction

In 2015, the World Health Organization (WHO) outlined global targets to eliminate Hepatitis C Virus (HCV) as a public health threat by 2030 [1]. It did so in the context of the recent progress of promising novel direct-acting antivirals (DAAs), drugs that were shown to have unprecedented trial capacity of curing HCV [2–4]. However, recent analysis of 45 high-income countries has demonstrated that just nine are on pace to achieve these goals, and that most are not even projected to reach them by 2050—with the USA falling into this latter group [5]. This sobering reality is cause to look outside of the efficacy of DAAs in a vacuum, and calls for analyzing the entire HCV care cascade—i.e., the model used to evaluate patient retention across sequential stages of care from screening to cure. As a disease that disproportionally afflicts persons who are traditionally less engaged in the healthcare system, it is necessary to explore and sort the social determinants of health that affect patients as they undergo each step of the HCV treatment process. In doing so, providers will be able to identify and address those distinct unmet needs—out of the many that often co-occur—which have the greatest impact at specific points along the HCV care cascade.

The majority of HCV infections occur in marginalized and stigmatized patient populations: people who inject drugs (PWID), homeless persons, justice-involved persons, and persons with HIV co-infection. More than half of persons with injection drug use (IDU) will contract HCV within 5 years of starting injecting, resulting in a prevalence of over 60% among people who inject drugs [6–8]. Other non-injection substance use disorders (SUDs), like alcohol use disorder and cocaine use disorder, are also highly prevalent in the HCV-infected population and often co-occur with IDU [9]. Further, a large HCV burden exists among persons who are homeless or unstably housed, housed in neighborhoods with poor access to care, and persons with serious mental illness [10–14]. Incarceration history, or justice involvement, has also been shown to be a negative predictor of SVR, and prison populations carry a disproportionate share of HCV-infected persons [15]. Moreover, insurance changes and terminations while on treatment are associated with poorer adherence and lower likelihood of achieving cure [16]. The demands of living with multiple intersecting stressors can affect linkage to treatment, engagement, and adherence to HCV therapy, ultimately interfering with the patient maintaining HCV treatment as a priority and resulting in negative outcomes [17].

In the wake of treatment innovations, such as DAAs and improved access to community-based care for SUD and HCV treatment in non-specialized settings, there has been significant progress and evidence of comparable cure rates being achieved in people who inject drugs as compared to other patient groups in clinical trials [18–21]. Despite these advances, there remains evidence of suboptimal treatment adherence and clinical outcomes among some groups of HCV patients, including persons with active IDU or HIV co-infection, as a complex array of clinical and psychosocial barriers continues to deter treatment success in real world cohorts of these historically vulnerable populations [22–26].

While adherence to a full course of antiviral treatment is important for achieving viral response, an underappreciated step of the HCV care cascade is the assessment and confirmation of a sustained virologic response (SVR). The ultimate goal of HCV treatment, SVR is achieved if the HCV viral load is undetectable by sensitive polymerase chain reaction (PCR) testing 12 weeks after HCV treatment completion. The assessment of SVR not only identifies viral relapse or reinfection but further allows for patient education on relapse prevention, repeated or future liver cancer screening, and the importance of linkage to primary care for this often vulnerable group of patients.

HCV treatment requires daily adherence for a usual period of 2–3 months, with follow-up to determine SVR after an additional 12 weeks. Many studies have examined viral suppression as an outcome, but more granular assessment of patient retention along the HCV care cascade is necessary to determine and address barriers to adherence and treatment completion that deter successful outcomes. Achievement of SVR12 is the ultimate goal of HCV treatment and it should always be included as the final step in the HCV care cascade. Despite the many advances made in the DAA era and access to HCV treatment in the primary care setting, interdisciplinary strategies and integrated models addressing underlying structural and individual social factors have not advanced sufficiently to achieve World Health Organization treatment elimination goals for HCV [5, 27]. Therefore, we sought to assess factors associated with HCV treatment completion and post-treatment SVR12 outcome assessment in our large urban cohort of people treated for HCV in our primary care and harm reduction-focused clinic.

## Methods

### Program description

In this study, we assessed potential predictors of incomplete treatment and failure to attend SVR assessment in treatment completers (a previously unstudied outcome) in a retrospective urban primary-care cohort of HCV-infected persons treated in the Respectful and Equitable Access to Comprehensive Healthcare (REACH) Program at Mount Sinai Hospital. Located in the East Harlem neighborhood of New York City, a neighborhood with historically high HCV prevalence, the REACH Program provides medical, mental health, social work and patient navigation services to HCV-infected persons with complex medical and psychosocial profiles [28]. The program addresses all steps in the HCV cascade of care, including community-based testing, linkage to care, retention in care, treatment readiness assessment, and reducing barriers to successful treatment completion and adherence. Building on work that has shown high rates of patients accessing medication and treatment, the program utilizes patient navigators working with a nurse and one specialty pharmacy to manage the prior authorization (PA) process, lessening the administrative burden on physicians and patients [29]. At each visit, including SVR12 visits, patients have access to a team of physicians, nurse practitioners, social workers, and mental health care providers.

Since 2011, the REACH program has employed the Psychosocial Readiness Evaluation and Preparation for Hepatitis C Treatment (PREP-C; prepc.org). This structured clinical assessment was developed to provide a standardized method for evaluating a patient’s readiness to begin HCV treatment and helps providers identify psychosocial factors that can potentially interfere with treatment adherence; thus highlighting opportunities to intervene in order to improve these areas of functioning and educate about HCV infection and treatment prior to treatment initiation. The nine areas of PREP-C assessment are: motivation, information, medication adherence, self-efficacy, social support and stability, alcohol and substance use, psychiatric stability, energy level, and cognitive functioning. A summary score is assigned for each domain according to a programmed algorithm, informing providers which areas “Could Be Improved”, which areas are “Satisfactory”, and which areas “Need Further Evaluation”.

### Study cohort

All patients underwent a comprehensive initial medical visit with a corresponding electronic medical record template and a PREP-C assessment allowing for the systematic collection of all data points. A total of 329 patients who initiated treatment between December 11, 2014 to March 2, 2018 met these criteria and were included in the study. At baseline, once initiating treatment, patients were scheduled to return for assessment at weeks 2, 4, 8, 12, 16, and 24 for 12-week courses of treatment and weeks 2, 4, 8, 12, and 20 for 8-week courses of treatment.

### Data collection

Approval from the Institutional Review Board of the Mount Sinai School of Medicine was obtained to conduct a retrospective chart review of program databases and the medical records of all patients in the REACH program who began HCV medications. We extracted data from both electronic medical records (EMR) and PREP-C assessment on all 329 patients. From the EMR we collected demographics, insurance type, HCV treatment history, laboratory values reflecting liver staging, presence of diabetes and other comorbidities. We collected information on history of and current psychiatric status and illicit substance use, housing status, and incarceration history (within or outside the past 5 years as a proxy for justice involvement) from the PREP-C assessment. We ascertained the degree of liver fibrosis from the following sources, in ranked order (if a higher level of data was not available, i.e., liver biopsy, the next source was used): liver biopsy, liver transient elastrography, Fibrometer (noninvasive fibrosis index) or FIB-4 score [30]. Our primary outcome, completing treatment, was assessed in all patients and was defined as completing the prescribed course of DAAs. Our other outcome, returning for SVR, was defined as returning for viral load testing at least once 12-week post-treatment completion regardless of SVR status. Of note, while defined as simply returning for viral load testing, the vast majority of SVR12 visits included critical health components such as reinfection education, cancer screening, and linkage to primary care. The datasets used during the current study are available from the corresponding author on reasonable request.

### Data analysis

We compared baseline characteristics of the cohort by treatment initiation status using the *t* test for normally distributed continuous variables and the *χ*^2^ test for categorical variables, as appropriate. We were interested in several different domains as predictors of completing treatment and returning for SVR. We therefore chose variables that represented these domains from our univariable analyses a priori and fit a multivariable logistic regression model that included demographics (age, sex, and race/ethnicity), liver staging results, presence of current or lifetime psychiatric illness, presence of current or lifetime substance use, insurance type, diabetes status, housing status, incarceration history, having another comorbidity (i.e., cardiovascular disease, chronic kidney disorder, etc.), and current methadone use. We evaluated model fit statistics but did not modify the model construction based on these factors as we planned to use the model to test our conceptual model that the chosen factors may have contributed to the outcomes. All analyses were performed with STATA 13 (Stata Corporation, College Station, TX).

## Results

Complete data was collected from a cohort of 329 patients who started HCV treatment in the REACH Program at Mount Sinai Hospital between 2014 and 2018. Our cohort was largely non-white (81% Table 1) and had a high prevalence of history of substance use disorders (93%) and psychiatric illness (84%). From the original cohort of 329 patients who started HCV treatment, 302 (92%) completed treatment. Patients who did not complete treatment were less likely to have stable housing in univariable and multivariable comparisons (Table 2: adjusted odds ratio [OR] for treatment completion: 0.3; 95% confidence interval [CI]: 0.1–0.9; other multivariate results not otherwise shown). Patients who completed treatment did not differ demographically in terms of age, gender, ethnicity, insurance type from those who did not (all *P* > 0.05). Additionally, there was no difference in prevalence of history of or current psychiatric illness, history of or current substance use, methadone maintenance therapy, HIV coinfection, diabetes, incarceration history, or cirrhosis between those who completed treatment and those who did not (all *P* > 0.05).

**Table 1:** Baseline demographics and disease characteristics

Total *n* (%)	Treatment completion (*n* = 329)		*P *value	Return for SVR 12 test (*n* = 302)		*P* value
	Yes	No		Yes	No	
	302 (92)	27 (8)		226 (75)	76 (25)	
*Age*						
Mean	53.5	53.2	0.882	54.3	51.3	0.059
Range	20–77	36–82		24–77	20–70	
20–30	19 (6)	0 (0)	0.36	11 (5)	8 (11)	0.076
31–50	85 (28)	11 (41)		62 (27)	23 (30)	
51–70	187 (62)	15 (56)		142 (63)	45 (60)	
71–82	11 (4)	1 (4)		11 (5)	0 (0)	
*Gender* *n* (%)			0.862			0.53
Male, *n* (%)	219 (73)	20 (74)		166 (73)	53 (70)	
Female, *n* (%)	83 (27)	7 (26)		60 (27)	23 (30)	
*Race/ethnicity*			0.71			0.854
African American or Black, *n* (%)	71 (24)	4 (15)		54 (24)	17 (22)	
Hispanic, *n* (%)	117 (39)	13 (48)		85 (37)	32 (42)	
White, *n* (%)	58 (19)	5 (19)		43 (19)	15 (20)	
Other, *n* (%)	56 (19)	5 (19)		44 (19)	12 (16)	
Cirrhotic, *n* (%)	58 (19)	6 (22)	0.704	50 (22)	8 (11)	**0.026**
*Insurance type*		0.525		**0.033**		
Private, *n* (%)	12 (4)	2 (7)		11 (5)	1 (1)	
Medicaid, *n* (%)	207 (69)	21 (78)		145 (64)	62 (82)	
Medicare, *n* (%)	43 (14)	1 (4)		39 (17)	4 (5)	
Medicare + medicaid, *n* (%)	39 (13)	3 (11)		30 (13)	9 (12)	
Uninsured, *n* (%)	1 (0)	0 (0)		1 (0)	0 (0)	
Uses medicaid, *n* (%)	246 (81)	24 (89)	0.33	175 (77)	71 (93)	**0.002**
HIV coinfection, *n* (%)	8 (3)	2 (7)	0.168	4 (2)	4 (5)	0.101
Diabetes, *n* (%)	47 (16)	3 (11)	0.537	42 (19)	5 (7)	**0.013**
Other comorbidity, *n* (%)	182 (60)	14 (52)	0.393	146 (64)	36 (47)	**0.008**
Current substance use, *n* (%)	90 (30)	10 (37)	0.434	65 (29)	25 (33)	0.496
Current psychiatric Illness, *n* (%)	82 (27)	10 (37)	0.273	66 (29)	16 (21)	0.167
Lifetime substance use, *n* (%)	282 (93)	25 (93)	0.129	208 (92)	74 (97)	0.106
Lifetime psychiatric Illness, *n* (%)	253 (84)	24 (89)	0.141	186 (82)	67 (88)	0.231
*Housing status*			**0.003**			0.777
Stable, *n* (%)	243 (80)	15 (56)		181 (80)	62 (82)	
Unstable, *n* (%)	59 (20)	12 (44)		45 (20)	14 (18)	
Incarceration history			0.166			0.831
< 5 Years ago, *n* (%)	64 (21)	10 (37)		48 (21)	16 (21)	
> 5 years ago, *n* (%)	159 (53)	11 (41)		117 (52)	42 (55)	
Never, *n* (%)	79 (26)	6 (22)		61 (27)	18 (24)	
Methadone use, *n* (%)	196 (65)	20 (74)	0.336	138 (61)	58 (76)	**0.016**

Statistically significant *p*-values (*p*-value < 0.05) were bolded

**Table 2:** Logistic regression models: (1) treatment completion; and (2) return for SVR12 testing

Predictor	Treatment completion		Return for SVR 12	
	OR	95% CI	OR	95% CI
Age	1	0.9–1.0	1	0.97–1.03
Female	0.8	0.3–2.3	0.6	0.3–1.2
*Ethnicity*				
White	Reference			
Black or African-American	1.1	0.2–4.8	0.7	0.3–1.8
Hispanic	0.6	0.2–2.1	0.7	0.3–1.4
Other	0.8	0.2–3.3	0.9	0.3–2.3
*Uses medicaid*	0.7	0.2–2.5	**0.3**	**0.1**–**0.7**
Cirrhosis	0.8	0.3–2.3	2	0.9–4.8
Diabetes	1.4	0.3–5.5	2.5	0.9–7.2
Other comorbidity	1.2	0.5–2.9	1.5	0.8–2.7
Current substance use	0.8	0.3–2.2	1	0.5–1.9
Current psychiatric Illness	0.9	0.4–2.2	1.6	0.8–3.3
Lifetime substance use	2.1	0.3–13.7	0.5	0.1–2.5
Lifetime psychiatric Illness	1.3	0.2–6.6	1.6	0.5–4.8
*Incarceration history*				
Never	Reference			
< 5 years ago	0.6	0.2–2.1	1.3	0.5–3.2
> 5 years ago	1.1	0.3–3.5	0.9	0.4–2.0
Methadone use	0.6	0.2–2.1	0.5	0.2–1.1
Unstable housing status	**0.3**	**0.1**–**0.9**	1.2	0.6–2.4

*Statistically significant findings bolded

We then evaluated the characteristics of the 302 patients who completed treatment and assigned the mutually exclusive outcome of either returning for SVR testing at least 12 weeks later or not (SVR12). Among those completing treatment, 226 (75%) returned for SVR12 assessment and 76 (25%) did not. Timing of SVR assessment ranged from 12 to 113 weeks, with 13 weeks being the median time period between treatment completion and returning for their post-12-week viral load testing. In univariable comparisons patients who returned for SVR were more likely to have cirrhosis (*P* = 0.03), diabetes (*P* = 0.01), or another major comorbidity (such as CHF, CKD, etc.; *P* = 0.008). Furthermore, returning patients were less likely to have Medicaid (*P* = 0.002) and less likely to receive methadone maintenance therapy (*P* = 0.02). The two groups did not differ across any of the other variables, including common demographics, history of or current psychiatric illness, history of or current substance use, housing stability, incarceration history, and HIV coinfection (all *P* > 0.05). In our multivariable regression model, Medicaid as primary insurance was the only independent predictor of not returning for SVR testing (OR = 0.3; 95% CI 0.1–0.7).

Of the 226 patients who completed treatment and returned for SVR12 testing, 221 (98%) achieved SVR. Of the 27 patients who did not complete treatment, 10 did eventually return for viral load testing at least 12 weeks after their anticipated treatment end date. Of those 10 patients, 7 achieved SVR (70%; *P* < 0.001 for comparison with treatment completers).

Of note, univariate testing did not demonstrate any relationship between any of the nine PREP-C domain scores and treatment completion or return for SVR12 testing; thus these data were excluded from Table 1 and logistic regression models.

## Discussion

This study evaluated sociodemographic predictors of treatment completion and return for post-treatment viral load testing in a patient cohort that was predominantly non-white, with a high prevalence of incarceration history, current and past psychiatric illness, current and past substance use, and unstable housing. Patients were treated in an urban primary care clinic caring for patients with SUDs and HCV. We found that the major independent predictor of treatment completion was housing status. Lack of follow-up for confirmation of viral “cure” was common in our cohort. We identified several factors associated with non-adherence to post-treatment testing (at 12 weeks or greater), with the major predictor being Medicaid as primary insurance, a proxy for socioeconomic status. We therefore find that social determinants of health are more strongly associated with adherence of key steps of the HCV treatment cascade than demographic or comorbid factors.

In our urban HCV treatment program, we found that 92% of patients completed HCV treatment, a proportion similar to other comparable patient cohorts with high rates of alcohol and substance use disorders, psychiatric illness, and on opiate agonist therapy [19, 31, 32]. We found unstable housing to be the sole independent predictor of patients not completing treatment. This finding is aligned with other studies demonstrating unstable housing to be a barrier in HCV care. Housing instability has been associated with non-adherence to HCV therapy, increased HCV acquisition, increased injecting frequency, and reduced access to addiction and HCV treatment resources [33–35]. On a larger scale, unstable housing has been identified as a social determinant of health that is more predictive of ill health than an individual’s behavior related to diet, physical exercise, smoking and alcohol consumption [36, 37]. Indeed, housing instability has been shown to increase an individual’s usage of emergency department and hospital services [33]. Exploring further, housing is strongly tied to other important social determinants of health, such as access to quality education and employment opportunities, access to quality nutritious foods, and exposure to violence [38]. Programs directed at supporting patients’ housing stability have shown to decrease emergency department visits, resulting in less strain on the healthcare system [37, 39].

Other studies have found alcohol use, illicit drug use, and mental illness to be associated with HCV treatment non-completion; however these findings were not replicated in our study [21, 24, 40]. The unique structure of our program, which operates with an emphasis on educating and supporting our patients with alcohol and substance use disorders based on structured psychosocial assessment, may play a role in the lack of association of these exposures with treatment non-adherence. Indeed, past studies have demonstrated the benefits in patient experience, healthcare engagement, and overall health outcomes when healthcare is provided in a manner that destigmatizes injection drug use [41–43]. Further, the REACH clinic’s all-encompassing focus on medical, psychological, and social aspects of a patient’s health incarnates and validates prior calls for multidisciplinary harm reduction care models [10, 29, 44, 45]. Our findings of high treatment completion amongst these at risk populations in the REACH model of integration of primary care and harm reduction for PWID and HCV treatment is encouraging yet makes clear that additional novel strategies to educate and support patients with unstable housing to complete treatment are needed.

In the care of patients with HCV, the assessment of treatment completion and confirmation of SVR is integral to ensure treatment success, provide post-treatment counseling and reinforce prevention of reinfection. This time in HCV care serves as an important opportunity to leverage ascertainment of HCV cure as a motive to continue efforts to address other comorbidities and optimize patient overall health [46]. Our study is the first we are aware of to look at the psychosocial and medical predictors of patients returning for SVR assessment. Of our patients who completed treatment, 75% returned for confirmatory viral load testing at least 12 weeks later. This suboptimal return rate is similar to others reported in similar patient cohorts [47–50]. Notably, there were multiple significant univariable associations between returning for testing and having comorbidities outside of HCV that required more participation in healthcare services; that is, patients who returned for testing were more likely to have had diabetes, cirrhosis, and any “other” comorbidity than patients who did not return. The strongest independent predictor, Medicaid as sole insurance, again suggests that socioeconomic determinants may have stronger impact on HCV treatment outcomes in urban HCV populations than medical, psychiatric or even substance use related factors. This is consistent with past research on associations of HCV health determinants with Medicaid status; persons with Medicaid have been less likely to be aware of their HCV status; had more steps in the prior authorizations process; and had longer wait times to receive prescriptions [29, 51, 52].

Medicaid itself serves as a proxy for other social determinants of health, as research has demonstrated that most medicaid beneficiaries lack in at least one social need—i.e., adequate income, food, housing, utilities, etc.[53]. These unmet needs have been associated with an increased number of chronic conditions as well as decreased physical functioning and higher mortality [53–55]. Recognizing this, the Centers for Medicare and Medicaid Services modernized its operations in 2016 to promote practices that look beyond clinical care and to address patients’ social needs [56]. While the roll-out of this “upstream” approach will vary on a state-by-state basis, the hope is that the expanded program will help bridge the gap between overall health and health care. Other more “downstream” strategies like patient navigation services and integrated harm reduction models of care—which provide patients with access to assistance programs—have been shown to improve the HCV care cascade [29, 57]. In this study, having Medicaid insurance appears to capture a risk for becoming disengaged in health care even after successfully completing HCV treatment.

Our study was bolstered by the richness of information, both psychosocial and medical, that we were able to obtain from our program model and utilization of the PREP-C. The detailed clinical picture obtained from this information guided our comprehensive treatment strategies and is a major factor in the high rate of treatment completion that we observed. With that said, our model of care, which specializes in providing primary-care-based HCV treatment for patients who inject drugs using a harm reduction approach, may limit the generalizability of our findings. As active efforts continue to expand HCV treatment into a more generalized primary care setting, is our hope that harm reduction techniques employed by the REACH clinic do so as well. Given that we used a liberal definition of returning for SVR12—from 12 to 113-week post-treatment completion—we assigned those who did complete treatment but did not return for SVR12 testing to be lost to care; however, it is possible that they may have established care elsewhere. Also, the possibility exists that a patient could have completed treatment with us, been reinfected and subsequently retreated outside of our health system—and thus outside of our knowledge—only to return for SVR12 at a later date within our 113-week range. Furthermore, our data is from a state that provides access to HCV care to patients regardless of liver staging, psychiatric illness, alcohol or substance use, insurance status, or HCV reinfection.

## Conclusion

In a retrospective study of 329 majority non-white patients with high prevalence of psychiatric illness and substance use disorders treated in an urban primary care clinic specialized in providing HCV care to people who use drugs between 2014 and 2018, we found that housing instability and Medicaid insurance were associated with lower rates of HCV treatment completion and return for treatment outcome assessment, respectively. These findings can inform future HCV care models, as novel strategies are created and implemented to ensure that at-risk patients not only complete treatment but remain engaged with the healthcare system post-treatment completion.

## Data Availability

The datasets used and/or analyzed during the current study are available from the corresponding author on reasonable request.

## Abbreviations

HCV: Hepatitis C virus
SVR: Sustained virologic response
DAA: Direct-acting antiviral
PWID: People who inject drugs
IDU: Injection drug use
SUD: Substance use disorder
PCR: Polymerase chain reaction
REACH: Respectful and equitable access to comprehensive healthcare
HepCAP: Hepatitis C patient assistance program
PA: Prior authorization
PREP-C: Psychosocial readiness evaluation and preparation for hepatitis C treatment
SVR12: Sustained virologic response testing 12 weeks after treatment completion
OR: Odds ratio
CI: Confidence interval
